# Predictive Value of Mean Platelet Volume in Patients Undergoing Conventional Dialysis and Online Hemodiafiltration

**DOI:** 10.7759/cureus.90334

**Published:** 2025-08-17

**Authors:** Diana D Nenova, Yanko G Yankov

**Affiliations:** 1 Second Department of Internal Disease, Medical University "Prof. Dr. Paraskev Stoyanov", Varna, BGR; 2 Clinic of Nephrology and Dialysis, University Hospital "St. Marina", Varna, BGR; 3 Clinic of Maxillofacial Surgery, University Hospital "St. Marina", Varna, BGR; 4 Department of General and Operative Surgery, Medical University "Prof. Dr. Paraskev Stoyanov", Varna, BGR

**Keywords:** death, dialysis, mean platelet volume, online hemodiafiltration, platelet, predictor, prognosis, ratio, reactivity, thrombogenesis

## Abstract

Introduction

Patients with end-stage renal disease (ESRD) experience significantly lower survival rates compared to the general population. Despite advances like online hemodiafiltration (OL-HDF), these patients still face high mortality, primarily due to cardiovascular disease (CVD). Mean platelet volume (MPV), an inexpensive and readily available marker of platelet activity and thrombogenesis, may help predict cardiovascular risk (CVR) and poor outcomes, but remains underutilized and under-researched in dialysis patients.

The aim of our study is to investigate MPV and the MPV-to-platelet count ratio (MPR) as potential markers for CVR and mortality in patients undergoing conventional hemodialysis and online hemodiafiltration, while also assessing how these therapies impact platelet activity to help improve patient outcomes.

Materials and methods

This retrospective analysis was carried out at the Clinic of Nephrology and Dialysis at the University Hospital "St. Marina" in Varna, Bulgaria. The study spanned two years, from January 2020 through December 2021, during which the medical records and routine laboratory results of 41 patients who met the study inclusion criteria were reviewed. The study evaluated platelet parameters - MPV, relative platelet count (PLT), and MPR - and their impact on clinical outcomes in two patient groups undergoing OL-HDF and hemodialysis (HD). Outcomes included dialysis adequacy and nutritional status indices, anemia control parameters, and annual mortality. The relationship between platelet parameters and these clinical indicators, as well as their prognostic value, was analyzed.

Results

The findings show that OL-HDF Group had notably higher levels of dialysis adequacy and nutritional status markers, along with elevated hemoglobin levels obtained using a significantly reduced erythropoietin dose. Regarding the platelet indices studied, a significantly lower MPV, higher PLT, and lower MPR ratio were observed compared to patients on HD. A strong positive correlation between MPV and erythropoietin dose was found in both groups (p<0.05). Data analysis showed that the relative risk of death in patients with MPV >10.5 fL was 5.59 times higher.

Discussion

PLT contribute not only to thrombogenesis but also to chronic inflammation via thrombokine release. While larger MPV indicates increased platelet reactivity, its predictive value in chronic kidney disease (CKD) remains debated. Some suggest elevated MPV reflects hypervolemia rather than reactivity. Studies have shown a negative correlation between MPV and both hospitalization frequency/duration and clinical outcomes in CKD patients.

Conclusion

Higher MPV values are significantly linked to clinical deterioration based on the type of dialysis, though not to the dialysis dose itself. Convective therapies lower mortality risk by improving clinical outcomes, enhancing middle molecule clearance, and reducing platelet reactivity and endothelial dysfunction. MPV and MPR are cost-effective, accessible markers for CVR stratification, aiding timely clinical decisions and interventions.

## Introduction

Patients with end-stage renal disease (ESRD) have significantly lower survival rates than the general population. According to the 2023 United States Renal Data System (USRDS) report, while relative mortality decreased by 29% between 2001 and 2016, overall mortality remains alarmingly high, with a 19.3% increase reported in 2023. The most recent available figure for deaths is from 2018, when 142340 cases were recorded. Despite advances in technologies such as online hemodiafiltration (OL-HDF), which enhances convective clearance of medium-sized molecules and improves clinical outcomes, these patients still face high mortality rates, primarily due to cardiovascular disease (CVD) [1-3].

Multiple randomized controlled trials (RCTs) and meta-analyses have evaluated the potential survival benefits of OL-HDF compared with conventional high-flux hemodialysis (HD). The landmark Estudio de Supervivencia de Hemodiafiltración Online (ESHOL) trial demonstrated a 30% reduction in all-cause mortality and a 55% reduction in infection-related mortality in patients receiving high-volume OL-HDF (>23 L/session) [4]. The more recent CONVINCE (Comparison of High-Dose Hemodiafiltration Versus High-Flux Hemodialysis) trial confirmed these findings, showing a significant reduction in all-cause and infection-related mortality, with a trend toward lower cardiovascular mortality [5]. Individual patient data meta-analyses and large observational cohorts have further supported that the mortality benefit of OL-HDF is strongly volume-dependent, with higher convection volumes associated with improved outcomes [6-8]. However, some trials using lower convective doses, such as the Turkish OL-HDF study (~17 L/session), did not observe significant survival advantages [9]. These results suggest that while OL-HDF can offer substantial clinical benefits, especially in reducing cardiovascular and infection-related deaths, its effectiveness depends heavily on achieving adequate convection volumes and addressing the advanced comorbidities of the ESRD population.

Unlike the general population, where cardiovascular mortality has been steadily decreasing, this improvement has not been mirrored in dialysis patients. Roughly 50% of deaths in this group are still attributed to CVD. This persistent disparity is largely due to the heavy burden of comorbidities and the older age profile of HD patients. Around 40% have diabetes, the average age is approximately 65 years, over 20% are older than 75, and many already exhibit significant left ventricular hypertrophy at the start of dialysis [3,10,11].

Risk factors such as hyperlipidemia, inadequate blood pressure control, excessive ultrafiltration rate, hyperphosphatemia associated with secondary hyperparathyroidism, vascular calcification, endothelial dysfunction, and increased thrombotic activity in a uremic setting should not be underestimated [3,10,11].

Mean platelet volume (MPV) serves as a valuable, though frequently underutilized, marker of platelet function and activation. Larger platelets, which exhibit greater metabolic and enzymatic activity, are notably more reactive and may act as indicators of increased cardiovascular risk (CVR) and poor outcomes in patients [12]. However, despite its affordability and easy availability, MPV remains under-researched as a predictor of clinical outcomes in individuals undergoing dialysis.

The aim of our study is to investigate MPV and the MPV-to-platelet count ratio (MPR) as potential markers for CVR and mortality in patients undergoing HD and OL-HDF. Evidence for the use of MPV and MPR as predictors of cardiovascular events and mortality has been reported in both dialysis and nondialysis populations, where elevated MPV and higher MPR have been associated with increased platelet reactivity, thrombotic risk, and worse survival outcomes. Additionally, the study seeks to assess the impact of both types of therapies on platelet activity, with a view to routinely implementing strategies in clinical practice that improve patient outcomes and survival.

## Materials and methods

This retrospective analysis was carried out at the Clinic of Nephrology and Dialysis at the University Hospital "St. Marina" in Varna, Bulgaria. The study spanned two years, from January 2020 through December 2021, during which the medical records and routine laboratory results of 41 patients who met the study inclusion criteria were reviewed. The study received ethical approval under protocol number 107/28.10.2021 from the Commission on Ethics of Research at the Medical University “Prof. Dr. Paraskev Stoyanov”, Varna, Bulgaria. This article was previously deposited in the institutional repository of the same university as part of Diana Nenova’s doctoral dissertation submitted in April 2022.

As inclusion criteria, participants were required to be at least 18 years old, have been on chronic hemodialysis for more than six months, exhibit minimal residual kidney function (defined as urine output less than 100 ml per day), be treated with low molecular weight heparin to standardize the results, be treated with a standardized antiplatelet regimen of aspirin at an equal dose, and have complete medical records.

Exclusion criteria included individuals under 18 years of age; those with malignancies, hematological disorders, or uncorrected iron deficiency; individuals with active bleeding from any source; patients receiving conventional heparin (to rule out heparin-induced thrombocytopenia); those on steroid or immunosuppressive therapy; individuals who had recently received granulocyte colony-stimulating factors or blood transfusions; individuals who have documented clinical and laboratory evidence of active infection; and patients with incomplete medical documentation.

As a part of the study, the medical records of 62 patients were reviewed, of which 47 met the study criteria. Out of these, 41 patients who fulfilled the inclusion and exclusion criteria were analyzed and statistically processed. Six patients, with an average age of 57.4±4.3 years (33.3% women and 66.7% men), were excluded from the cohort. Among them, four had a malignant condition, and two was on treatment with steroids, related to autoimmune or inflammatory diseases, which may affect the result for platelet count. The average age of excluded patients (57.4 years) was similar to the analyzed cohort, minimizing age-related bias. Importantly, baseline demographic and clinical characteristics of the analyzed 41 patients were carefully matched between HD and OL-HDF groups, ensuring internal comparability. While exclusion reduces sample size, it enhances the internal validity and specificity of the findings by eliminating major confounders.

The study population was divided into two groups: Group 1 (n=22), which underwent OL-HDF in post-dilution mode using high-flux F70 dialyzers and received a high convection volume (Qo>20 liters per session); and Group 2 (n=19), which received conventional HD with low-flux polysulfone Asahi dialyzers. Both groups were treated with a dialysis flow rate (Qd) of 500 ml/min and a blood flow rate (Qb) of 300±42 ml/min, with a mean total procedure time of 12±0.13 hours per week. The small variation in the duration of the procedures, as well as in the blood flow rate used, is a necessary condition for the comparability of the results. Dialysis was performed using Fresenius Medical Care machines from the 4008 and 5008 series (Fresenius Medical Care AG & Co., Bad Homburg, Germany).

The study evaluated platelet-related parameters in both groups - MPV, platelet count (PLT), and MPR - to determine their impact on clinical outcomes. These outcomes included measures of dialysis adequacy, such as single-pool Kt/V (spKt/V) and urea reduction ratio (URR%), nutritional indicators like normalized protein catabolic rate (nPCR) and serum albumin, and anemia management parameters such as hemoglobin (Hb) levels and average weekly doses of erythropoiesis-stimulating agents (ESA). Additionally, annual mortality rates were recorded for both groups, and the relationship between these rates and platelet parameters was analyzed to evaluate their prognostic value.

Laboratory assessments for complete blood count and biochemical parameters - including urea, creatinine, and serum albumin - were routinely conducted for all study participants following the guidelines established by the National Health Insurance Fund of the Republic of Bulgaria. Blood samples were collected under standardized conditions: hemoglobin measurements utilized blood drawn into K2EDTA tubes, while serum samples were obtained using vacutainers containing gel separators. These samples were centrifuged at 2500 g for 15 minutes (6-8 mL volume), and the resulting serum was used for the analysis of urea, creatinine, and albumin concentrations.

Hemoglobin concentration (g/L) was measured as part of the complete blood count using the colorimetric method with sodium lauryl sulfate on a 6-differential hematology analyzer (Sysmex XN1000, Siemens, Munich, Germany). The reference range for hemoglobin is 120-180 g/L, with target levels for patients with ESRD set between 110-120 g/L.

PLT count was determined using the electrical impedance method. MPV was automatically calculated by dividing the plateletcrit by the platelet count. PLT results were expressed in units of 10⁹/L. Additionally, MPR was calculated by dividing MPV by PLT, and reported as a numerical value. The reference range for PLT is 140-440x10⁹/L and for MPV is 6.0-10.0 fL.

Urea concentration (mmol/L) was measured using a coupled enzyme reaction involving glutamate dehydrogenase (GLDH) through a UV kinetic method on the ADVIA Chemistry 1800 system (Siemens), with a reference range of 3.2-8.2 mmol/L. Creatinine concentration (mmol/L) was assessed using the Jaffe kinetic method on the same analyzer, with a reference range of 44-115 mmol/L. Albumin levels (g/L) were determined by a colorimetric assay employing bromocresol green (BCG) as a selective dye, also using the ADVIA Chemistry 1800, with a reference range of 32-48 g/L.

To minimize the effects of recirculation and urea rebound, the stop-pump technique was applied before and/or after HD.

Blood samples for hemoglobin and albumin were routinely collected before the dialysis session to avoid interference from ultrafiltration (UF). MPV and PLT samples were obtained pre-dialysis, consistent with the timing for Hb and albumin sampling. Collecting these samples before the dialysis session minimizes the influence of dialysis-related platelet activation or sequestration, ensuring that the values reflect the patients’ baseline hematologic status. Samples for urea and creatinine were taken both pre- and post-dialysis to evaluate dialysis adequacy and nutritional status using indices such as spKt/V, URR%, and nPCR. The calculations for these parameters are outlined in Table 1.

**Table 1:** Mathematical methods catabolic rate; t: dialysis duration in hours (h); UF: ultrafiltration volume in liters (l); W: post-dialysis body weight in kg; ln: natural logarithm; Co: pre-dialysis urea nitrogen concentration; C: post-dialysis urea nitrogen concentration; Cn: pre-dialysis urea nitrogen concentration from the next hemodialysis; R: urea ratio (C/Co); ID: interdialytic interval in hours (h) spKt/V: single-pool Kt/V; URR%: urea reduction ratio; nPCR: nutritional indicators like normalized protein catabolic rate

Indicator	Formula
spKt/V	spKt/V=​​-ln(R-0.008xt)+[4-3.5xR]x0.55UF/W)
URR%	URR%=100×(1–C/Co)
nPCR	nPCR=0.22+0.36×(Cn-C)×24/ID

Data were analyzed using IBM SPSS Statistics for Windows, version 20.0 (IBM Corp., Armonk, NY, USA), running on the Windows 10 operating system (Microsoft Corp., Redmond, WA, USA). Descriptive statistics were employed to summarize quantitative variables as means and measures of variability, while qualitative variables were described using absolute and relative frequencies. For hypothesis testing, parametric tests such as the Student’s t-test were used, alongside non-parametric tests including the Chi-square test and Cramér’s V, Cohen's d test was used to indicate the effect size of the difference between two means. It quantifies the magnitude of the difference between the means of two groups, essentially showing how far apart the groups are in terms of standard deviations. Receiver operating characteristic (ROC) curve analysis was performed to evaluate the predictive performance of selected variables, and relative risk (RR) analysis was conducted to assess outcome likelihood in exposed groups. A p-value of less than 0.05 was considered statistically significant.

## Results

The baseline characteristics of the two groups are presented in Table 2. Both groups were comparable in terms of gender distribution, prevalence of diabetes and cardiovascular disease, underlying cause of ESRD, use of antihypertensive agents and aspirin, and dialysis vintage. The lack of significant differences supports the validity of comparing clinical outcomes between these groups without major concern for confounding by these baseline factors.

**Table 2:** Baseline demographic and clinical characteristics of the study groups This table summarizes the distribution of demographic variables, comorbidities, causes of end-stage renal disease (ESRD), medication use, and dialysis vintage for Group 1 and Group 2. Data are expressed as number (percentage) for categorical variables and mean±standard deviation for continuous variables. p-values are derived from Chi-square test for categorical variables, and independent samples t-tests for continuous variables. ESRD: end stage of renal disease; CVD: cardiovascular disease; n: number of studied patients

Characteristic	Group 1 (n=22)	Group 2 (n=21)	p-value	Interpretation
Sex, Male, n (%)	12 (54.5%)	11 (52.4%)	0.88	No significant difference
Diabetes, n (%)	11 (50%)	12 (57.1%)	0.58	No significant difference
CVD history, n (%)	12 (54.5%)	11 (52.4%)	0.89	No significant difference
Cause of ESRD: diabetes	11 (50%)	10 (47.6%)	0.83	No significant difference
Cause of ESRD: hypertension	6 (27.3%)	7 (33.3%)	0.67	No significant difference
Cause of ESRD: glomerulonephritis	5 (22.7%)	4 (19.0%)	0.74	No significant difference
Antihypertensives, n (%)	20 (90.9%)	19 (90.5%)	0.96	No significant difference
Aspirin 75 mg daily, n (%)	22 (100%)	21 (100%)	—	No difference
Dialysis vintage (years)	8.38±2.09	8.45±2.01	0.88	No significant difference

Table 3 presents the results of variation analysis and Student’s t-test comparing patients undergoing conventional HD and OL-HDF over the two-year study period. A statistically significant difference (p<0.05) was found between the two groups for most parameters, with the exception of serum albumin levels at the study’s conclusion (t=1.20289, p=0.238), where no significant difference was observed.

**Table 3:** Data from the variational analysis and Student's t-test for the two-year period of follow-up of the two studied groups spKt/V: single-pool Kt/V, dialysis delivered dose index; URR%: urea reduction ratio, dialysis delivered dose index; nPCR (g/kg/d): normalized protein catabolic rate, nutritional status index; Alb (g/l): serum albumin level; Hgb (g/l): hemoglobin level; ESA (UI/week): erythropoietin stimulating agent weekly dose; MPV (fL): mean platelet volume; PLT (x 10⁹/L): platelet count; MPR: mean platelet volume-to-platelet count ratio

Indicator (X±SD)	Group 1 (n=22)	Group 2 (n=19)	t-test	p-value
Age (years)	55.6±11.78	55.64±11.12	-0.738	0.465
spKt/V	1.83±0.25	1.37±0.08	7.54	<0.00001
URR %	79.21±3.56	71.86±2.99	6.36	<0.00001
nPCR (g/kg/d)	1.31±0.12	1.2±0.05	3.48	0.0024
Alb (g/l)	38.17±3.96	36.64±2.98	1.20289	0.238
Hgb (g/l)	112.15±6.86	104.14±4.73	3.64	<0.00001
ESA (UI/week)	6142±2080	9714±1749	-4.01	0.000162
MPV (fL)	9.61±0.86	11.43±0.62	-6.58974	<0.00001
PLT (x10⁹/L)	243.62±44.06	170.43±28.49	5.3369	<0.00001
MPR	0.04±0.07	0.08±0.01	-1.74251	0.045367

The findings indicate that Group 1 (OL-HDF) exhibited significantly better outcomes in terms of dialysis adequacy and nutritional status. Moreover, these patients achieved higher serum hemoglobin levels while requiring a significantly lower dose of erythropoietin.

Regarding the platelet indices studied, a significantly lower MPV, higher platelet count, and lower MPR ratio were observed in Group 1 compared to patients in Group 2.

Table 4 presents post-hoc statistical power calculations for the primary and secondary outcomes, comparing the OL-HDF and HD groups.

**Table 4:** Post-hoc statistical power analysis for study indicators spKt/V: single-pool Kt/V, dialysis delivered dose index; URR%: urea reduction ratio, dialysis delivered dose index; nPCR (g/kg/d): normalized protein catabolic rate, nutritional status index; Alb (g/l): serum albumin level; Hgb (g/l): hemoglobin level; ESA (UI/week): erythropoietin stimulating agent weekly dose; MPV (fL): mean platelet volume; PLT (x 10⁹/L): platelet count; MPR: mean platelet volume-to-platelet count ratio; n: number of studied patients

Indicator	Group 1 (n=22)	Group 2 (n=19)	Cohen’s d	Observed power (%)
spKt/V	1.83±0.25	1.37±0.08	2.40	100.0
URR (%)	79.21±3.56	71.86±2.99	2.22	100.0
nPCR (g/kg/d)	1.31±0.12	1.20±0.05	1.17	95.2
Alb (g/L)	38.17±3.96	36.64±2.98	0.43	27.0
Hgb (g/L)	112.15±6.86	104.14±4.73	1.34	98.7
ESA (UI/week)	6142±2080	9714±1749	1.85	99.99
MPV (fL)	9.61±0.86	11.43±0.62	2.40	100.0
PLT (×10⁹/L)	243.62±44.06	170.43±28.49	1.94	100.0
MPR	0.04±0.07	0.08±0.01	0.77	67.1

Cohen’s d effect sizes were calculated for each parameter, with interpretation thresholds of small (0.2), medium (0.5), and large (0.8+). Despite the modest sample size, most primary outcomes - such as spKt/V, URR, nPCR, hemoglobin, ESA dose, MPV, and platelet count - demonstrated large effect sizes and very high observed power (>95%). Albumin and MPR exhibited smaller effect sizes and correspondingly lower power, reflecting their status as secondary endpoints. These results confirm that the study was sufficiently powered to detect clinically meaningful differences in the main outcomes, reducing the likelihood of Type II errors and supporting the reliability of the observed effects.

**Figure 1:**
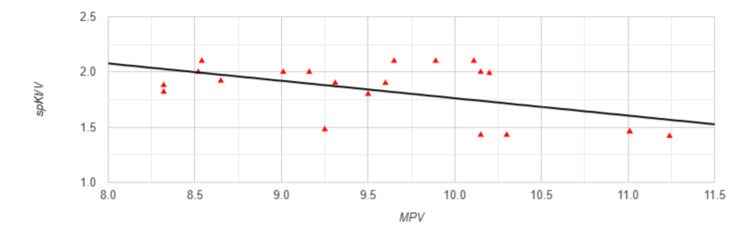
Correlation between MPV and spKt/V in Group 1 MPV: mean platelet volume; spKt/V: single-pool Kt/V, dialysis adequacy index

The Pearson correlation analysis revealed different types of correlations between MPV and the studied parameters in Group 1. A strong negative correlation was found between MPV and spKt/V (r=-0.5394, p=0.012) (Figure 1).

**Figure 2:**
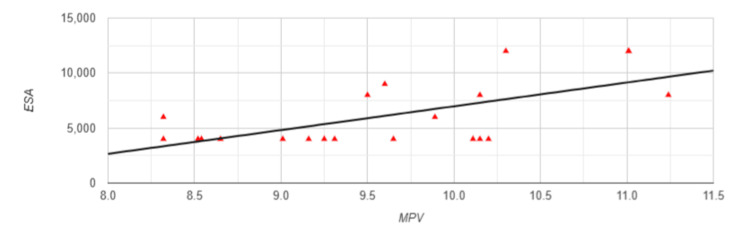
Correlation between MPV and ESA in Group 1 MPV: mean platelet volume; ESA: erythropoiesis-stimulating agents

Regarding the control of the anemic syndrome, a significant positive correlation with a strong relationship was observed between MPV and the administered erythropoietin dose (r=0.6444, p=0.002) (Figure 2). In contrast, the negative correlation found between MPV and hemoglobin levels did not reach statistical significance (r=-0.363, p=0.106).

No correlations were found between MPV and serum albumin levels or nPCR (p>0.05).

**Figure 3:**
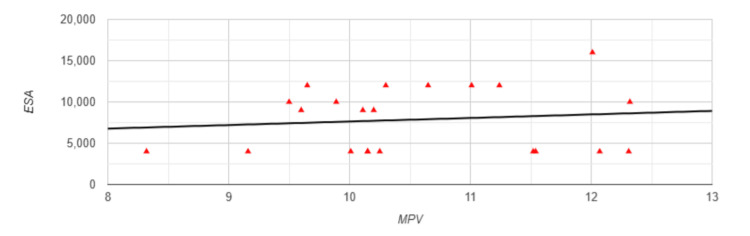
Correlation between MPV and ESA in Group 2 MPV: mean platelet volume; ESA: erythropoiesis-stimulating agents

Despite significantly higher MPV values in Group 2, which also showed notably lower values in parameters reflecting clinical outcomes, the correlations between MPV and the aforementioned indicators did not reach statistical significance (p>0.05). Аn exception is a medium positive correlation between MPV and ESA (r=0.471, p=0.027) (Figure 3).

The analysis also revealed significant differences in mortality rates during the observation period: 14.3% in Group 1 and 42.8% in Group 2 (χ²=13.147, Cramér’s V=0.513, p=0.001). Patients in Group 2 had a 3.6-fold higher risk of death compared to those in Group 1 (RR=3.59; 95% CI: 1.2531-10.2467, p<0.001). This increased mortality in Group 2 was associated with significantly elevated MPV and lower platelet counts.

The Chi-square test indicated a strong correlation between elevated MPV levels (MPV>10.5 fL) and mortality across both groups (χ²=19.240, Cramér’s V=0.439, p=0.001). Further analysis demonstrated that patients with higher MPV values had a 5.59-fold increased risk of death compared to those with MPV<10.5 fL (RR=5.59; 95% CI: 0.5979-46.8151, p<0.01). It should be noted that the relative risks reported above reflect different analyses: the 3.59-fold increase corresponds to the overall risk of mortality in HD patients compared to OL-HDF patients, while the 5.59-fold increase refers specifically to patients exceeding the MPV threshold and in the entire sample.

**Figure 4:**
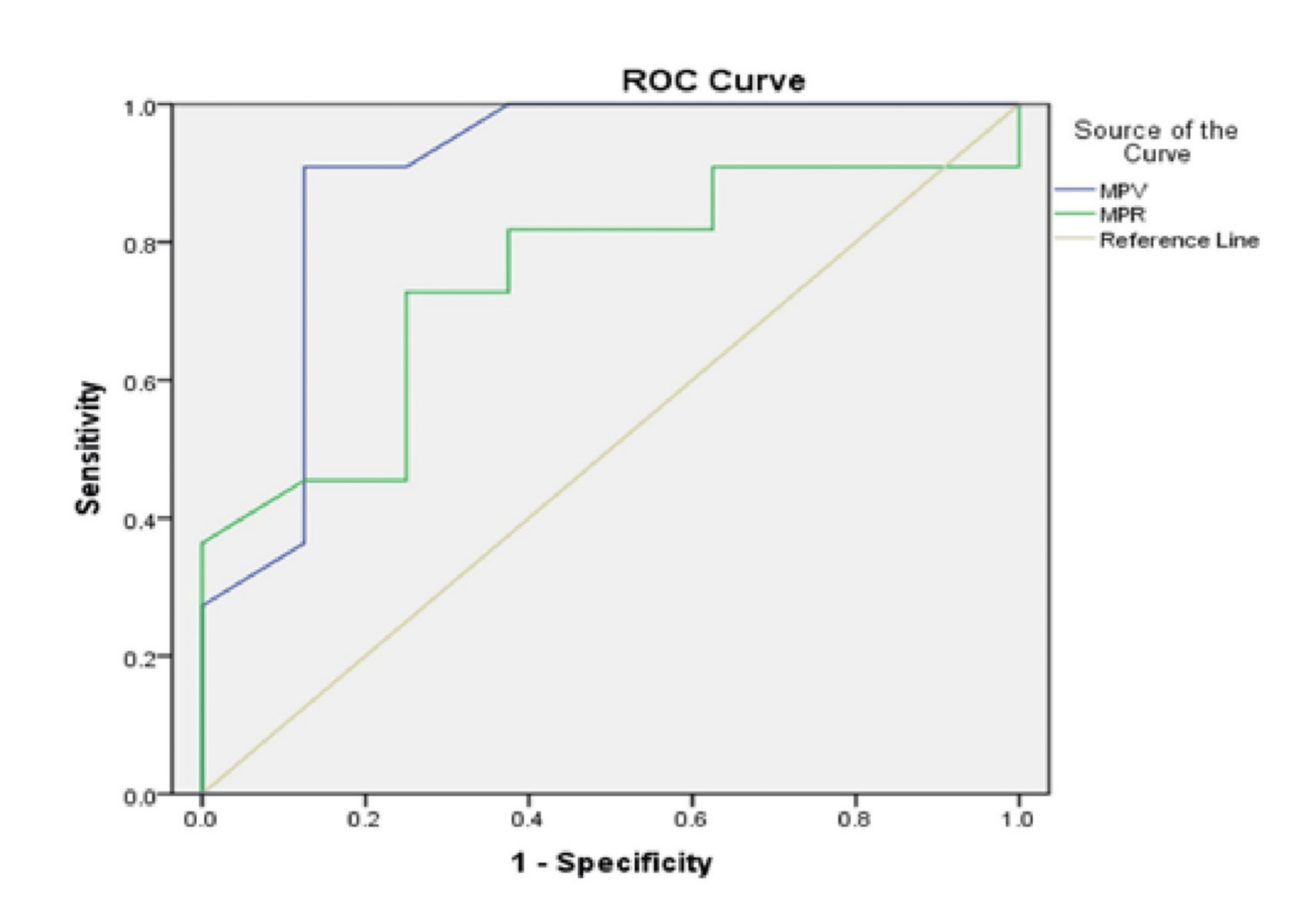
ROC curves for evaluating predictors of poor clinical outcomes in Group 1. AUC for MPV=0.898 (95% CI: 0.73-1.00, p=0.004), AUC for MPR=0.744 (95% CI: 0.51-0.97, p=0.076). ROC: receiver operating characteristic; blue line: mean platelet volume (MPV); green line: mean platelet volume-to-platelet count ratio (MPR); yellow line: reference line; AUC: area under curve

**Figure 5:**
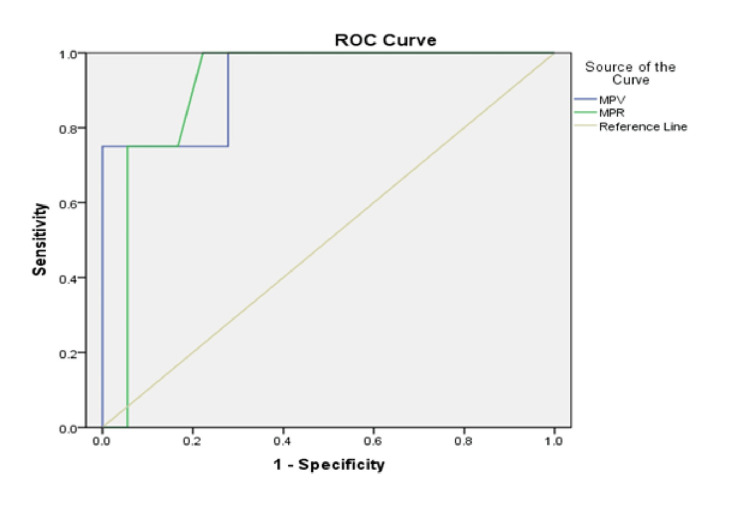
ROC curves for evaluating predictors of poor clinical outcomes in the hemodialysis Group 2. AUC for MPV=0.93 (95% CI: 0.794-1.00, p=0.008), AUC for MPR=0.91 (95% CI: 0.781-1.00, p=0.012). ROC: receiver operating characteristic; blue line: mean platelet volume (MPV); green line: mean platelet volume-to-platelet count ratio (MPR); yellow line: reference line; AUC: area under curve

Figure 4 and Figure 5 illustrate the ROC curves used to assess the predictive value of MPV and the MPR for mortality and clinical deterioration over the two-year study period. The ROC analyses differentiated modality-specific cut-off values.

ROC analysis in the HD group revealed that both MPV and MPR demonstrated strong negative predictive value, with areas under the curve (AUC) exceeding 0.9. MPV showed an AUC of 0.93 (95% CI: 0.794-1.00, p=0.008), while MPR yielded an AUC of 0.91 (95% CI: 0.781-1.00, p=0.012). The identified optimal cut-off values in this group were MPV>10.5 fL (sensitivity: 76.9%; specificity: 77.8%) and MPR>0.038 (sensitivity: 72.8%; specificity: 76.98%).

In the OL-HDF group, ROC analysis showed predictive significance only for MPV, with an AUC of 0.898 (95% CI: 0.73-1.00, p=0.004). The critical MPV threshold associated with clinical deterioration in this group was higher than 11.05 fL, corresponding to a sensitivity of 84.6% and a specificity of 66.7%. MPR in the OL-HDF group did not reach statistical significance, with an AUC of 0.744 (95% CI: 0.51-0.97, p=0.076).

## Discussion

Patients with chronic kidney disease (CKD), especially those with ESRD, are predisposed to chronic inflammation due to both increased production and decreased clearance of pro-inflammatory cytokines [3,13]. This creates conditions for endothelial dysfunction and thrombogenesis relatively early in the pathogenesis of CKD and predicts cardiovascular events and adverse outcomes in these patients. Significant changes have been documented in the reactivity, as well as the count and mean platelet volume of platelets in patients with CKD, particularly those on hemodialysis [14].

Platelets play a crucial role not only in thrombogenesis but also in maintaining chronic inflammation through the release of various thrombokines [15,16]. It is well established that larger MPV corresponds to increased platelet reactivity [17]. However, the predictive value of MPV in patients with CKD remains controversial. For instance, Bilen et al. found that elevated MPV in CKD patients is linked more to concomitant hypervolemia than to heightened platelet reactivity [18]. Similarly, a study by Ju et al. reported increased MPV values accompanied by a progressive decline in platelet count and platelet distribution width (PDW) [19]. Additionally, patients with diabetic nephropathy have been shown to exhibit higher MPV levels compared to the general population [20].

According to Balcioglu and Kirlioglu, MPV is negatively correlated with both the frequency and duration of annual hospitalizations, as well as clinical outcomes, in patients with CKD [21]. However, research remains limited regarding the role and variation of platelet parameters - especially MPV - across different stages of CKD.

Our results demonstrate an increased MPV accompanied by a significantly lower platelet count and elevated MPR in the HD group compared to patients undergoing OL-HDF. This relationship does not directly correlate with the better clinical outcomes observed in the OL-HDF group, which include higher dialysis adequacy, improved nutritional status, and better control of anemia [3].

Despite the strong negative correlation between MPV and delivered dialysis dose observed overall, this correlation was not found in the HD group. This outcome is likely due to multiple factors: the increased clearance of middle molecules during convective therapies - some of which are activators of thrombogenesis and chronic inflammation - and the use of high-flux membranes in OL-HDF. These membranes have significantly larger pore sizes, higher biocompatibility, and cause less platelet damage and activation.

Currently, there are no studies specifically investigating platelet parameters in OL-HDF. However, research by Sari et al. and Kim et al. confirms increased platelet reactivity and MPV, as well as a direct relationship between MPV and platelet count [21,22]. Similar findings have been reported in patients on peritoneal dialysis, supporting our results [23].

Of particular interest is the strong correlation found between MPV and the administered erythropoietin dose. Erythropoietin, as a growth factor, further increases platelet activity, and the use of higher doses to maintain target hemoglobin levels stimulates thrombogenesis. This effect, combined with one of the main side effects of ESA - worsening of arterial hypertension-significantly increases cardiovascular risk in these patients.

Our findings reveal a strong association between convective therapy and improved anemia management, as evidenced by significantly higher hemoglobin levels and a consistent reduction in the required dose of ESA compared to patients undergoing conventional HD [3]. This enhanced response may contribute to the observed reduction in platelet activation and the lower MPV seen in the OL-HDF group.

These results are consistent with previous reports from several small uncontrolled studies and a limited number of randomized trials, which have shown that convective therapies are associated with better anemia control. Improved outcomes have been noted particularly following the transition from low-flux hemodialysis (LF-HD) to OL-HDF using high-permeability, biocompatible membranes [3,24-26]. Similarly, Ok et al. (2013) reported a lower ESA requirement in the OL-HDF group, although the difference in hemoglobin levels did not reach statistical significance [3,9].

The mortality data observed during the study period suggest a potential survival benefit associated with convective therapies. A statistically significant difference in overall mortality was identified between the two groups, with patients in the conventional hemodialysis group exhibiting a 3.6-fold higher risk of death compared to those receiving OL-HDF (RR=3.59; 95% CI: 1.2531-10.2467, p<0.001). These findings support an association between convective modalities and improved patient survival outcomes [3].

On one hand, this is linked to improved clinical outcomes in the OL-HDF group, reflected by better dialysis clearance, nutritional status, and control of the anemia syndrome. On the other hand, it is associated with reduced thrombogenesis and platelet reactivity, expressed by lower MPV and MPR values and a significantly higher platelet count.

Our results demonstrate a strong association between mortality and high platelet volumes - MPV>10.5 fL (χ²=19.240, V=0.439, p=0.001). Patients with MPV above this threshold had a 5.59-fold higher relative risk of death, regardless of the type of therapy.

Data from the ROC curve analyses underline the strong predictive ability of platelet indices for mortality in both groups. For the HD group, this predictive value is very high for both MPV and MPR, with critical worsening thresholds at MPV>10.5 fL and MPR>0.038, and this is not directly dependent on clinical outcomes or achieved dialysis dose. For the convective therapy group, only MPV showed predictive value, with a critical threshold for worsening at MPV>11.05 fL, which is significantly higher compared to conventional dialysis.

The association between high MPV and increased mortality and poor prognosis is further supported by the study of Kim et al., which found that both baseline and time-dependent elevated MPV levels were linked to a progressively higher risk of death. In multivariate analyses, high MPV was associated with an increased mortality risk, while low MPV corresponded to a reduced risk, using normal MPV values as the reference [22].

Similar results were observed in a multicenter cross-sectional study involving 518 hemodialysis patients, where those with MPV in the highest quintile (>9.2 fL) faced an increased risk of coronary heart disease compared to patients in the lowest quintile (<7.2 fL). This association remained significant after adjusting for confounding factors such as age, smoking status, blood pressure, and various laboratory parameters [27].

In the general population, MPV is well established as a key predictor of thrombosis, thromboembolism, and adverse cardiovascular events, both primary and secondary. Elevated MPV levels have additionally been linked to a higher risk of ischemic stroke, greater brain injury volume, and increased early mortality in the post-stroke period [28,29].

Similar findings have been observed in dialysis patients. A study by Ma et al. demonstrated the strong predictive value of elevated MPV and MPR for acute ischemic stroke occurrence and subsequent mortality, showing high sensitivity and specificity [30]. These results align closely with our own findings.

Limitations of the study

Several limitations of this study should be acknowledged. First, the retrospective design and relatively small sample size, constrained by the limited availability of dialysis devices, may affect the generalizability of the results and preclude definitive causal conclusions. However, comparability of baseline values between the HD and OL-HDF groups was ensured by strict inclusion/exclusion criteria and matching on key demographic and clinical parameters. Second, the duration of follow-up was limited due to the significantly higher cost of consumables associated with OL-HDF, which is not reimbursed separately by the National Health Insurance Fund and was therefore conducted on a voluntary basis within the general budget. Third, although inflammatory markers such as CRP or IL-6 were not available for the entire cohort, all patients with documented clinical or laboratory evidence of infection were excluded from the latter. Finally, although ESA therapy is known to potentially affect platelet indices, ESA doses were measured, reported, and compared between groups to account for this effect. To mitigate these limitations, we carefully selected a retrospective period and implemented standardized protocols to ensure the representativeness and internal validity of our patient sample.

## Conclusions

Elevated levels of MPV and MPR are independently associated with an increased risk of mortality and endothelial dysfunction in the growing dialysis population. A significant relationship is established between higher MPV values and the risk of clinical deterioration depending on the type of dialysis therapy, but not directly related to the delivered dialysis dose. The results suggest that, in this cohort, the use of convective therapies was associated with lower mortality risk, potentially linked to improved clinical outcomes, enhanced clearance of middle molecules, and lower platelet reactivity and endothelial dysfunction compared to conventional dialysis. Platelet parameters MPV and MPR emerge as inexpensive and accessible tools for cardiovascular risk stratification, enabling clinicians to make rapid decisions and therapeutic interventions. Due to the limited number of studies in this area, further research is needed to elucidate the mechanisms involved and to confirm the diagnostic value of these parameters in order to improve patient prognosis.

## References

[1] Johansen KL, Gilbertson DT, Li S (2024). US Renal Data System 2023 annual data report: epidemiology of kidney disease in the United States. Am J Kidney Dis.

[2] Saran R, Robinson B, Abbott KC (2019). US Renal Data System 2018 annual data report: epidemiology of kidney disease in the United States. Am J Kidney Dis.

[3] Nenova D (2022). Adequacy of Dialysis Treatment and the Relationship With Achieved Quality of Life and Survival in Patients With Stage V Chronic Kidney Disease. https://eprints.mu-varna.bg/handle/nls/2076.

[4] Maduell F, Moreso F, Pons M (2013). High-efficiency postdilution online hemodiafiltration reduces all-cause mortality in hemodialysis patients. J Am Soc Nephrol.

[5] Blankestijn PJ, Bots ML (2023). Effect of hemodiafiltration or hemodialysis on mortality in kidney failure. Reply. N Engl J Med.

[6] Vernooij RW, Hockham C, Strippoli G (2024). Haemodiafiltration versus haemodialysis for kidney failure: an individual patient data meta-analysis of randomised controlled trials. Lancet.

[7] Canaud B, Morena M, Leray-Moragues H, Chalabi L, Cristol JP (2006). Overview of clinical studies in hemodiafiltration: what do we need now?. Hemodial Int.

[8] Mostovaya IM, Blankestijn PJ, Bots ML (2014). Clinical evidence on hemodiafiltration: a systematic review and a meta-analysis. Semin Dial.

[9] Asci G, Tz H, Ozkahya M (2013). The impact of membrane permeability and dialysate purity on cardiovascular outcomes. J Am Soc Nephrol.

[10] Assimon MM, Wenger JB, Wang L, Flythe JE (2016). Ultrafiltration rate and mortality in maintenance hemodialysis patients. Am J Kidney Dis.

[11] De Luca G, Santagostino M, Secco GG (2009). Mean platelet volume and the extent of coronary artery disease: results from a large prospective study. Atherosclerosis.

[12] Klovaite J, Benn M, Yazdanyar S, Nordestgaard BG (2011). High platelet volume and increased risk of myocardial infarction: 39,531 participants from the general population. J Thromb Haemost.

[13] Baigent C, Burbury K, Wheeler D (2000). Premature cardiovascular disease in chronic renal failure. Lancet.

[14] Boehme M, Kaehne F, Kuehne A (2007). Pentraxin 3 is elevated in haemodialysis patients and is associated with cardiovascular disease. Nephrol Dial Transplant.

[15] Sari O, Bashir AM (2022). Early change in platelet count and MPV levels of patients who received hemodialysis for the first time: Mogadishu Somalia experience. Int J Clin Pract.

[16] Morrell CN, Aggrey AA, Chapman LM, Modjeski KL (2014). Emerging roles for platelets as immune and inflammatory cells. Blood.

[17] Karolczak K, Soltysik B, Kostka T, Witas PJ, Watala C (2019). Platelet and red blood cell counts, as well as the concentrations of uric acid, but not homocysteinaemia or oxidative stress, contribute mostly to platelet reactivity in older adults. Oxid Med Cell Longev.

[18] Bilen Y, Cankaya E, Keles M (2014). Does decreased mean platelet volume predict inflammation in chronic renal failure, dialysis, and transplanted patients?. Ren Fail.

[19] Ju HY, Kim JK, Hur SM (2015). Could mean platelet volume be a promising biomarker of progression of chronic kidney disease?. Platelets.

[20] Turgutalp K, Özhan O, Akbay E (2014). Mean platelet volume and related factors in patients at different stages of diabetic nephropathy: a preliminary study. Clin Appl Thromb Hemost.

[21] Balcioglu YH, Kirlioglu SS (2020). C-reactive protein/albumin and neutrophil/albumin ratios as novel inflammatory markers in patients with schizophrenia. Psychiatry Investig.

[22] Kim S, Molnar MZ, Fonarow GC (2016). Mean platelet volume and mortality risk in a national incident hemodialysis cohort. Int J Cardiol.

[23] Chen J, Zhong Z, Li J (2022). Effect of mean platelet volume to platelet count ratio on mortality in peritoneal dialysis. Mediators Inflamm.

[24] Li Y, Wang Y, Lv J, Wang M (2013). Clinical outcomes for maintenance hemodialysis patients using a high-flux (FX60) dialyzer. Ren Fail.

[25] Locatelli F, Carfagna F, Del Vecchio L, La Milia V (2018). Haemodialysis or haemodiafiltration: that is the question. Nephrol Dial Transplant.

[26] Mostovaya IM, Grooteman MP, Basile C (2015). High convection volume in online post-dilution haemodiafiltration: relevance, safety and costs. Clin Kidney J.

[27] Henning BF, Zidek W, Linder B, Tepel M (2002). Mean platelet volume and coronary heart disease in hemodialysis patients. Kidney Blood Press Res.

[28] Greisenegger S, Endler G, Hsieh K, Tentschert S, Mannhalter C, Lalouschek W (2004). Is elevated mean platelet volume associated with a worse outcome in patients with acute ischemic cerebrovascular events?. Stroke.

[29] Pikija S, Cvetko D, Hajduk M, Trkulja V (2009). Higher mean platelet volume determined shortly after the symptom onset in acute ischemic stroke patients is associated with a larger infarct volume on CT brain scans and with worse clinical outcome. Clin Neurol Neurosurg.

[30] Ma L, Han Q, Sun F, Zhu K, Sun Q (2023). Mean platelet volume/platelet count ratio as a predictor of both incidence and prognosis of acute ischemic stroke in hemodialysis patients. Int J Gen Med.

